# CRISPR screens identify genes essential for *in vivo* virulence among proteins of hyperLOPIT-unassigned subcellular localization in *Toxoplasma*

**DOI:** 10.1128/mbio.01728-24

**Published:** 2024-07-31

**Authors:** Yuta Tachibana, Miwa Sasai, Masahiro Yamamoto

**Affiliations:** 1 Department of Immunoparasitology, Research Institute for Microbial Diseases, Osaka, Japan; 2 Laboratory of Immunoparasitology, WPI Immunology Frontier Research Center, Osaka, Japan; 3 Department of Immunoparasitology, Center for Infectious Disease Education and Research, Osaka University, Osaka, Japan; Stanford University, Stanford, California, USA

**Keywords:** *Toxoplasma*, CRISPR screen, hyperLOPIT-unassigned virulence factors

## Abstract

The research field to identify and characterize genes essential for *in vivo* virulence in *Toxoplasma gondii* has been dramatically advanced by a series of *in vivo* clustered regularly interspaced short palindromic repeats (CRISPR) screens. Although subcellular localizations of thousands of proteins were predicted by the spatial proteomic method called hyperLOPIT, those of more than 1,000 proteins remained unassigned, and their essentiality in virulence was also unknown. In this study, we generated two small-scale gRNA libraries targeting approximately 600 hyperLOPIT-unassigned proteins and performed *in vivo* CRISPR screens. As a result, we identified several genes essential for *in vivo* virulence that were previously unreported. We further characterized two candidates, TgGTPase and TgRimM, which are localized in the cytoplasm and the apicoplast, respectively. Both genes are essential for parasite virulence and widely conserved in the phylum Apicomplexa. Collectively, our current study provides a resource for estimating the *in vivo* essentiality of *Toxoplasma* proteins with previously unknown localizations.

**IMPORTANCE:**

*Toxoplasma gondii* is a protozoan parasite that causes severe infection in immunocompromised patients or newborns. *Toxoplasma* possesses more than 8,000 genes; however, the genes essential for *in vivo* virulence were not fully identified. The apicomplexan parasites, including *Toxoplasma*, developed unique organelles that do not exist in other model organisms; thus, determining the subcellular location of parasite proteins is important for understanding their functions. Here, we used *in vivo* genetic screens that enabled us to investigate hundreds of genes in *Toxoplasma* during mouse infection. We screened approximately 600 parasite proteins with previously unknown subcellular localizations. We identified many novel genes that confer parasite virulence in mice. Among the top hits, we characterized two genes essential for *in vivo* virulence, TgGTPase and TgRimM, which are widely conserved in the phylum Apicomplexa. Our findings will contribute to understanding how apicomplexans adapt to the host environment and cause disease.

## INTRODUCTION

An obligate intracellular parasite, *Toxoplasma gondii*, is an important human and animal pathogen that causes life-threatening toxoplasmosis (1). *Toxoplasma* has been the model apicomplexan parasite owing to its easiness of *in vitro* cultivation, genetic tractability, *in vivo* disease model in mice, and various biological assays to investigate the function of the genes (2). As unicellular eukaryotes that adapt to intracellular parasitism, apicomplexans have developed unique secretory organelles (e.g., micronemes, rhoptries, dense granules), which are absent in other well-studied eukaryotic cell systems (3). These secretory organelles are key features of the apicomplexans and discharge various types of proteins that are essential for invasion, replication, and egress (3). Likewise, the apicoplast is a unique non-photosynthetic plastid-like organelle and essential for apicomplexans’ metabolism (fatty acid, heme, iron-sulfur cluster, and isoprenoid synthesis) (4). Therefore, uncovering the subcellular localizations of parasite proteins is crucial to understand the protein functions and parasite pathogenesis mechanism.

Although the parasite protein localization is typically revealed by cell biological methods, such as classical immunofluorescence assay (IFA), immuno-electron microscopy, and, recently, ultrastructure expansion microscopy (5, 6), the parasite spatial proteome has been largely unknown. Recent studies elegantly addressed the issues in *Toxoplasma* (7) and *Cryptosporidium* (8) by a spatial proteomic method called hyperLOPIT (9), yielding plenty of knowledge on parasite subcellular proteome. In a previous study using hyperLOPIT on *Toxoplasma* (7), 3,832 proteins were identified in the tachyzoite stage; however, 1,198 of them are not assigned to any subcellular niche by a method called t-augmented Gaussian mixture models—maximum *a posteriori* (TAGM-MAP) with high confidence and classified as hyperLOPIT-unassigned subcellular localizations. Moreover, the functions of these genes mostly remained to be elucidated.

In addition to classical forward and reverse genetics, *in vitro* or *in vivo* clustered regularly interspaced short palindromic repeats (CRISPR) genetic screens have been used to assess the essentiality of the parasite genes in the lytic cycles or virulence (10–15). We previously performed *in vivo* CRISPR screens using small-scaled guide RNA (gRNA) libraries based on hyperLOPIT-assigned localizations (e.g., rhoptries, dense granules, nucleus, endoplasmic reticulum [ER], Golgi apparatus, cytosol, apicoplast, and mitochondrion) and identified novel secreted and non-secreted virulence factors (14). However, the essentiality of hyperLOPIT (TAGM-MAP)-unassigned proteins in virulence remains mostly unknown.

In this study, we generated two small-scaled gRNA libraries targeting genes encoding hyperLOPIT (TAGM-MAP)-unassigned proteins and performed *in viv*o CRISPR screens in mice. We identified several novel genes that contribute to the parasite’s virulence. Among the top hits, we further focused on two candidates, TgGTPase (TGGT1_277840) and TgRimM (TGGT1_321310). TgGTPase is a guanosine triphosphatase (GTPase) that localizes in the parasite cytosol, and TgRimM is a novel apicoplast-resident protein possessing a ribosome maturation factor M (RimM) domain and a C2H2-type zinc finger domain. Both genes are widely conserved among the apicomplexans, and gene deletions led to the *in vitro* and *in vivo* growth defect and the complete loss of virulence in mice, suggesting that TgGTPase and TgRimM are essential for the parasite adaptation to the *in vitro* and *in vivo* environment. Overall, our current study provides a clue for the research community to estimate the *in vivo* requirements of proteins with previously unknown localizations.

## RESULTS

### *In vivo* CRISPR screening targeting proteins of unassigned subcellular localization

**Fig 1:**
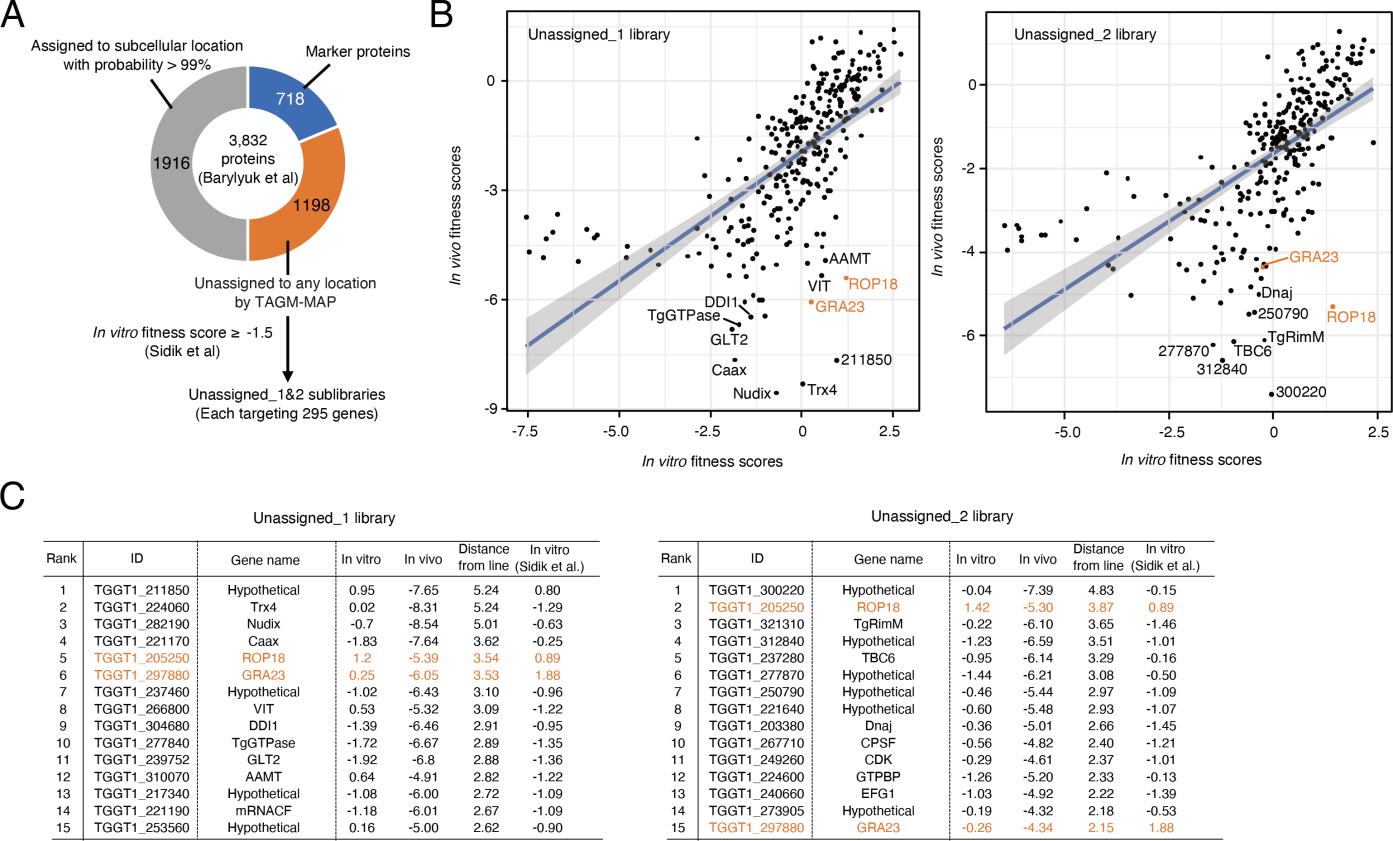
*In vivo* CRISPR screening targeting proteins with hyperLOPIT-unassigned localization. (**A**) Schematic of sublibraries targeting hyperLOPIT-unassigned proteins. (**B**) Scatterplots showing *in vitro* and *in vivo* fitness scores from Unassigned_1 (left) and Unassigned_2 (right) libraries, respectively. ROP18 and GRA23 are labeled in orange as control genes. (**C**) Ranking tables for top 15 *in vivo* fitness-conferring genes in each library ordered by the distance from the regression line.

To identify genes necessary for *Toxoplasma* survival in mice, we utilized a previously established *in vivo* CRISPR screen platform using type I strain and C57BL/6 mice (14). In the hyperLOPIT of *Toxoplasma* (7), 3,832 proteins were identified in the tachyzoite stage. Among them, 718 proteins were marker proteins for subcellular localization. The authors have assigned 1,916 proteins to one of subcellular locations with a localization possibility above 99% by TAGM-MAP. The remaining 1,198 proteins were not assigned to any subcellular location with sufficient reliability and classified as “unassigned.” Among 1,198 TAGM-MAP-unassigned proteins, we excluded genes with *in vitro* fitness scores less than −1.5 in previously published genome-wide screens to reduce the size of the gRNA library (10). Finally, we generated two gRNA libraries (Unassigned_1 and Unassigned_2 libraries), each targeting 295 genes (Fig. 1A). Transfected RHΔhxgprt parasites were selected with pyrimethamine in Vero cells for four passages to generate a pooled mutant population (*in vitro* sample). The parasite mutant pools were used to infect C57BL/6 mice with 10^7^ parasites each by injection into the footpad. After 7 days post-infection, parasites were recovered from the spleen and expanded in Vero cells for one passage (*in vivo* sample). The gRNA sequences were amplified by PCR from the input library and the genomic DNA from the parasites of *in vitro* and *in vivo* samples. The gRNAs were sequenced by next-generation sequencing to determine their relative abundance. We calculated *in vitro* and *in vivo* fitness scores of each gene as the average log_2_ fold change of guide abundances between conditions. We analyzed the screen results from both sublibraries to assess the reproducibility (Tables S1 and S2). The *in vitro* essential and dispensable controls were successfully separated, with lower scores for the essential genes and higher scores for the dispensable genes (Fig. S1A). The correlations between our *in vitro* fitness scores and the genome-wide *in vitro* fitness scores in human foreskin fibroblasts (HFF) were high (*r* = 0.78 and 0.76, respectively) (Fig. S1B) (10). High reproducibility of *in vivo* fitness scores was observed between independent infections in mice (*r* = 0.75 ± .05 and 0.68 ± 0.07, respectively) (Fig. S1C). These data demonstrated that our *in vitro* and *in vivo* CRISPR screens using Unassigned_1 and Unassigned_2 libraries were highly reproducible.

To identify genes that confer parasite *in vivo* fitness during infection in mice, we compared *in vitro* and *in vivo* fitness scores (Fig. 1B; Tables S1 and S2). To rank the candidate genes, we calculated the distance of each gene from the regression line (Fig. 1C). As reported previously (14), the *in vivo* essential controls ROP18 and GRA23 showed negative *in vivo* fitness scores and ranked highly (Fig. 1C). This analysis highlighted both previously identified and unidentified genes. For instance, the top hits from Unassigned_1 library contain hypothetical protein (TGGT1_211850), thioredoxin (Trx4: TGGT1_224060), NUDIX hydrolase (Nudix: TGGT1_282190), CAAX metallo endopeptidase (Caax: TGGT1_221170), vacuolar iron transporter (VIT: TGGT1_266800), DNA damage inducible protein 1 (DDI1: TGGT1_304680), TgGTPase (TGGT1_277840), GLT2 (TGGT1_239752), apical annuli methyltransferase (AAMT: TGGT1_310070), and mRNA cleavage factor family protein (mRNACF: TGGT1_221190). It has been reported that Trx4 is localized in parasitophorous vacuole (PV) (16). VIT mainly localizes to the plant-like vacuolar compartment (PLVAC) and regulates parasite iron metabolism (17). DDI1 is one of the components of the parasite ubiquitin–proteasome system and is localized in the cytoplasm and nucleus (18). TgGTPase was identified as an interactor of the Nd complex, which facilitates rhoptry exocytosis (19). GLT2 is a glucosyltransferase and required for parasite disaccharide metabolism (20). AAMT was identified as an apical annuli component (21). The top hits from Unassigned_2 library also contain several hypothetical proteins (TGGT1_300220, TGGT1_312840, TGGT1_277870, TGGT1_250790, TGGT1_221640, and TGGT1_273905), ribosome maturation factor RimM domain-containing protein (TgRimM: TGGT1_321310), TBC6 (TGGT1_237280), DnaJ domain-containing protein (Dnaj: TGGT1_203380), CPSF A subunit region protein (CPSF: TGGT1_ 267710), and rRNA-processing protein EFG1 (EFG1: TGGT1_240660). Although TGGT1_300220 was also shown to be one of the top hits in another *in vivo* CRISPR screen (15), no further investigation was performed. TBC6 is one of the TBC domain-containing proteins and localized in the ER or cytoplasmic vesicles (22, 23). TGGT1_273905 localizes to the cytoplasm (24). Although some candidates (Trx4, VIT, and DDI1) were previously reported to be required for parasite virulence (16–18), none of the other top hits have been assessed regarding virulence so far. To elucidate the mechanisms of these genes for *in vivo* fitness, we compared our *in vivo* screen results with those of gamma interferon (IFN-γ)-activated macrophages (Fig. S1D) (25). This comparison highlighted some of our screen hits (such as Nudix, Caax, and TGGT1_211850), which may function in IFN-γ-dependent mechanisms.

### Identification of novel genes that contribute to parasite virulence in mice

**Fig 2:**
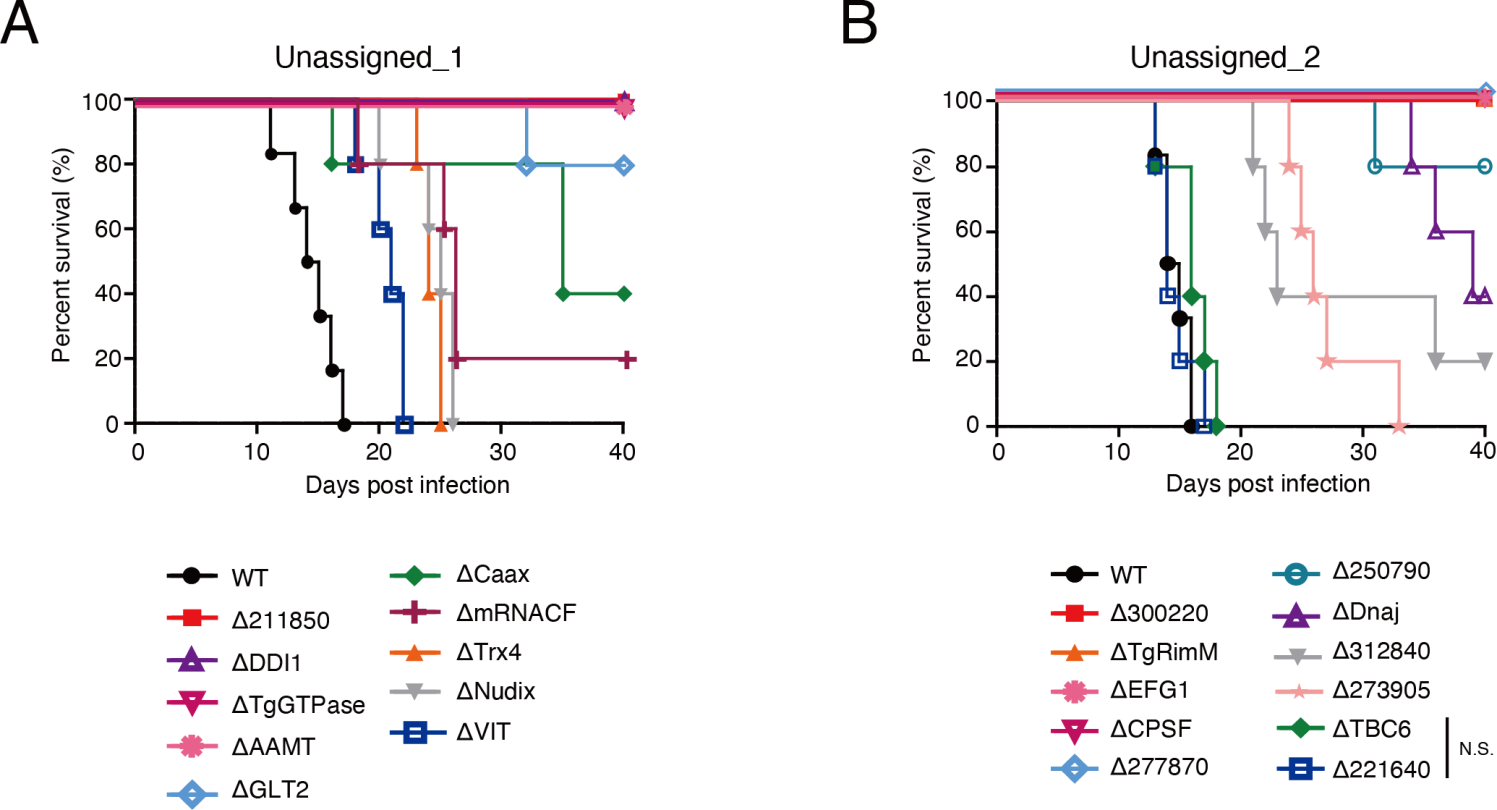
Validation of candidate genes that affect parasite virulence. (**A**) C57BL/6 mice were inoculated into the footpad with 10^3^ tachyzoites of the indicated knockout strains from the unassigned_1 library. WT (*n* = 6), ΔTGGT1_211850 (*n* = 5), ΔDDI1 (*n* = 5), ΔTgGTPase (*n* = 5), ΔAAMT (*n* = 5), ΔGLT2 (*n* = 5), ΔCaax (*n* = 5), ΔmRNACF (*n* = 5), ΔTrx4 (*n* = 5), ΔNudix (*n* = 5), ΔVIT (*n* = 5). (**B**) C57BL/6 mice were inoculated into the footpad with 10^3^ tachyzoites of the indicated knockout strains from the unassigned_2 library. WT (*n* = 6), ΔTGGT1_300220 (*n* = 5), ΔTgRimM (*n* = 5), ΔEFG1 (*n* = 5), ΔCPSF (*n* = 5), ΔTGGT1_277870 (*n* = 5), ΔTGGT1_250790 (*n* = 5), ΔDnaj (*n* = 5), ΔTGGT1_312840 (*n* = 5), ΔTGGT1_273905 (*n* = 5), ΔTBC6 (*n* = 5), ΔTGGT1_221640 (*n* = 5). N.S., not significant (log-rank test).

We generated individual knockout strains to examine whether these top-hit genes affect parasite virulence (Fig. S2A and B). WT mice were locally infected with these mutant strains into the footpad, and the survival rates were monitored. We chose footpad infection rather than intraperitoneal infection because our previous study revealed that the former is more suitable for assessing virulence in using RH strain (14) (Fig. 2A and B). Among the candidates from Unassigned_1 library, all mice survived in the infection of ΔTGGT1_211850, ΔDDI1, ΔTgGTPase, or ΔAAMT parasites. Other tested mutants also showed reduced virulence (ΔGLT2, ΔCaax, ΔmRNACF, ΔTrx4, ΔNudix, and ΔVIT). Among the candidates from Unassigned_2 library, all mice survived in the infection of ΔTGGT1_300220, ΔTgRimM, ΔEFG1, ΔCPSF, or ΔTGGT1_277870 parasites. ΔTGGT1_250790, ΔDnaj, ΔTGGT1_312840, or ΔTGGT1_273905 parasites showed reduced virulence. WT mice infected with ΔTBC6 and ΔTGGT1_221640 succumbed in a time course similar to those infected with WT parasites. Collectively, these data demonstrated that our *in vivo* CRISPR screens targeting hyperLOPIT (TAGM-MAP)-unassigned proteins highlighted known and novel genes essential for *in vivo* virulence.

### TgGTPase is a widely conserved GTPase that contributes to virulence

**Fig 3:**
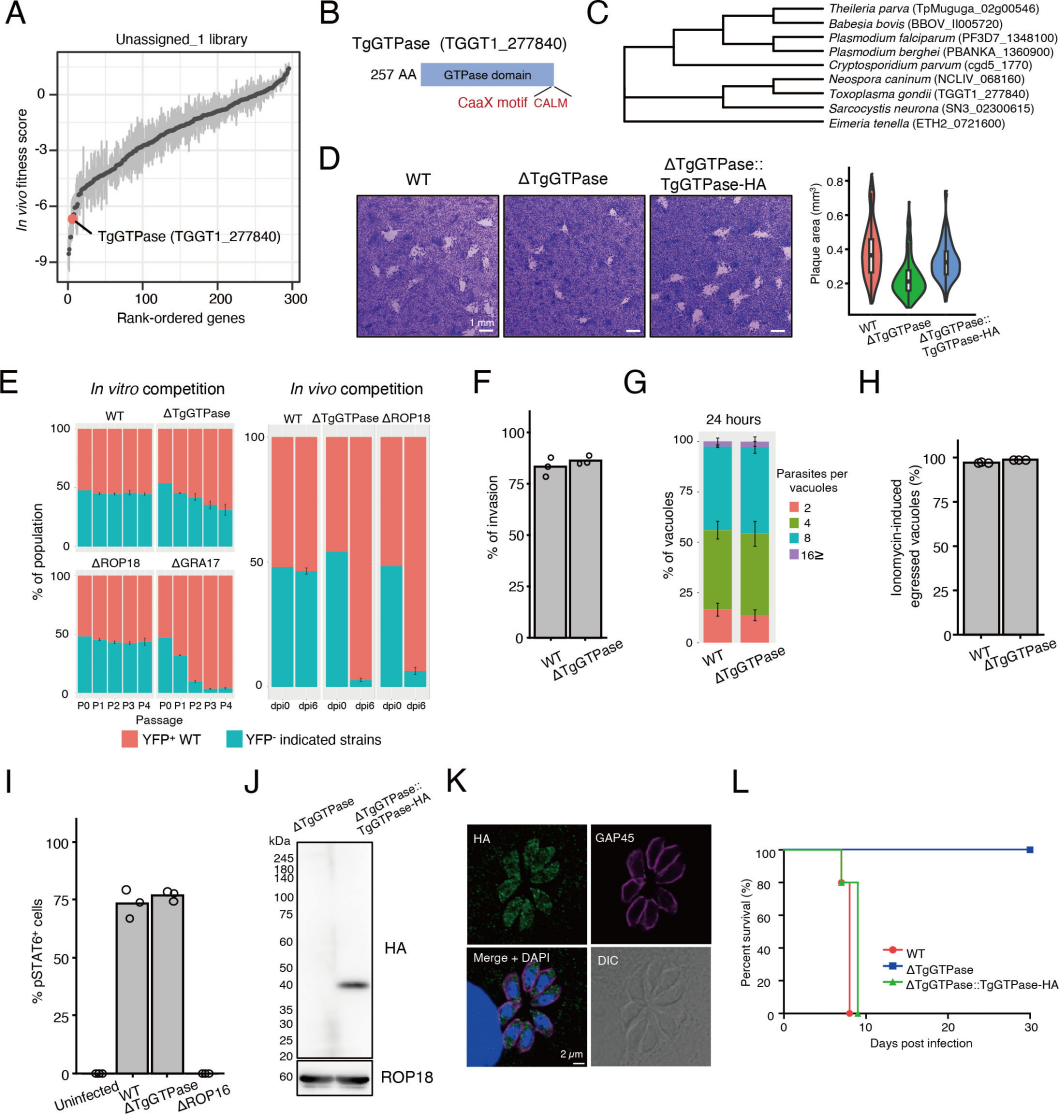
TgGTPase is a cytosolic GTPase that contributes to parasite virulence. (A) Rank-ordered plot of genes from Unassigned_1 library. (B) Schematic of putative domains in TgGTPase. (C) Phylogenetic tree of selected TgGTPase homologs across the apicomplexan phylum. (D) Plaque images and areas of indicated strains. (E) *In vitro* and *in vivo* competition assay of indicated strains. (F) Invasion assay of ∆TgGTPase and WT. (G) Intracellular replication assay of ∆TgGTPase and WT. (H) Induced egress assay of ∆TgGTPase and WT. (I) ROP16-mediated STAT6 phosphorylation assay of indicated strains. (J) Western blot analysis of TgGTPase-HA. (K) Immunofluorescence images of ΔTgGTPase::TgGTPase-HA showing cytosolic localization of TgGTPase-HA (green). GAP45 (magenta) stains the inner membrane complex of the parasites. (L) Survival curves of C57BL/6 mice intraperitoneally infected with 10^3^ tachyzoites of WT (*n* = 5), ΔTgGTPase (*n* = 5), and ΔTgGTPase::TgGTPase-HA (*n* = 5). Data are representative of two (D, E, F, G, and H) and three (I, J, and K) independent experiments.

Among the top-ranking candidates from Unassigned_1 library, we chose to characterize TgGTPase further (Fig. 3A; Table S1). TgGTPase is a GTPase that is broadly conserved across the phylum Apicomplexa (Fig. 3B and C).

We performed plaque assays to examine whether deletion of TgGTPase affects *in vitro* growth. We found that ∆TgGTPase parasites formed smaller plaques compared to WT parasites (Fig. 3D), suggesting that TgGTPase is important for *in vitro* growth. To assess the importance of TgGTPase in fitness, we performed competition assays in *in vitro* culture (Fig. 3E). ∆TgGTPase parasites showed a relatively mild competition defect in *in vitro* culture compared to WT or ∆ROP18 parasites, but not as severe as ∆GRA17 parasites, which are known to have an *in vitro* growth defect (26). We next performed competition assays in *in vivo* infection (Fig. 3E). We found that ∆TgGTPase parasites were significantly outcompeted by WT parasites in mice similar to ∆ROP18 parasites, which are known to have an *in vivo* growth defect (27–29). Our results suggest that TgGTPase is also important for *in vivo* growth. To investigate each step of the lytic cycle, we assessed parasite invasion, intracellular replication, egress, attachment, and gliding motility. We found that TgGTPase deletion did not affect those events (Fig. 3F through H; Fig. S3A and B). Since TgGTPase is reported as an interactor of the Nd complex (19), we next assessed whether TgGTPase deletion affects rhoptry secretion. We measured rhoptry discharge by assessing phosphorylation of STAT6 induced by ROP16 (30) and recruitment of Irgb6 to parasitophorous vacuole membrane (PVM) that is blocked by ROP18 (31). We did not find significant differences between ∆TgGTPase and WT parasites in both assays (Fig. 3I; Fig. S3C through E). Therefore, it is likely that TgGTPase does not have an essential function in the secretion of ROP16 or ROP18.

To study the function of TgGTPase, we complemented the ΔTgGTPase strain with C-terminal HA-tagged TgGTPase expressed from the tubulin promoter that we will refer to as “ΔTgGTPase::TgGTPase-HA” (Fig. S3F). ΔTgGTPase::TgGTPase-HA parasites restored normal plaque formation (Fig. 3D). Western blot showed the band of TgGTPase-HA at approximately 40 kDa (Fig. 3J). TgGTPase-HA showed a cytoplasmic localization by IFA (Fig. 3K). To validate the essentiality of TgGTPase on pathogenesis, we infected C57BL/6 mice intraperitoneally with 10^3^ tachyzoites of the WT, ∆TgGTPase, and ΔTgGTPase::TgGTPase-HA parasites and assessed mouse survival (Fig. 3L). All the mice intraperitoneally infected with ∆TgGTPase parasites survived. By contrast, ΔTgGTPase::TgGTPase-HA parasites fully restored virulence as well as WT parasites.

We realized that the C-terminal amino acid sequence of TgGTPase (CALM) is a CaaX motif, which is a sign of prenylation (Fig. 3B). TgGTPase was also identified as one of the candidates of prenylated protein by proteomic analysis (32). Thus, tagging at the C-terminus might interfere with the prenylation and localization of TgGTPase. Therefore, we newly generated N-terminally HA-tagged complemented strains that we refer to as “ΔTgGTPase::HA-TgGTPase” (Fig. S3F). We found the N-terminally tagged HA-TgGTPase also localized in the cytosol by IFA (Fig. S3G), agreeing with a previous report (32). These results suggest that TgGTPase is a cytoplasmic protein essential for virulence.

### TgRimM is a novel apicoplast-resident protein possessing C2H2 zinc finger that contributes to virulence in mice

**Fig 4:**
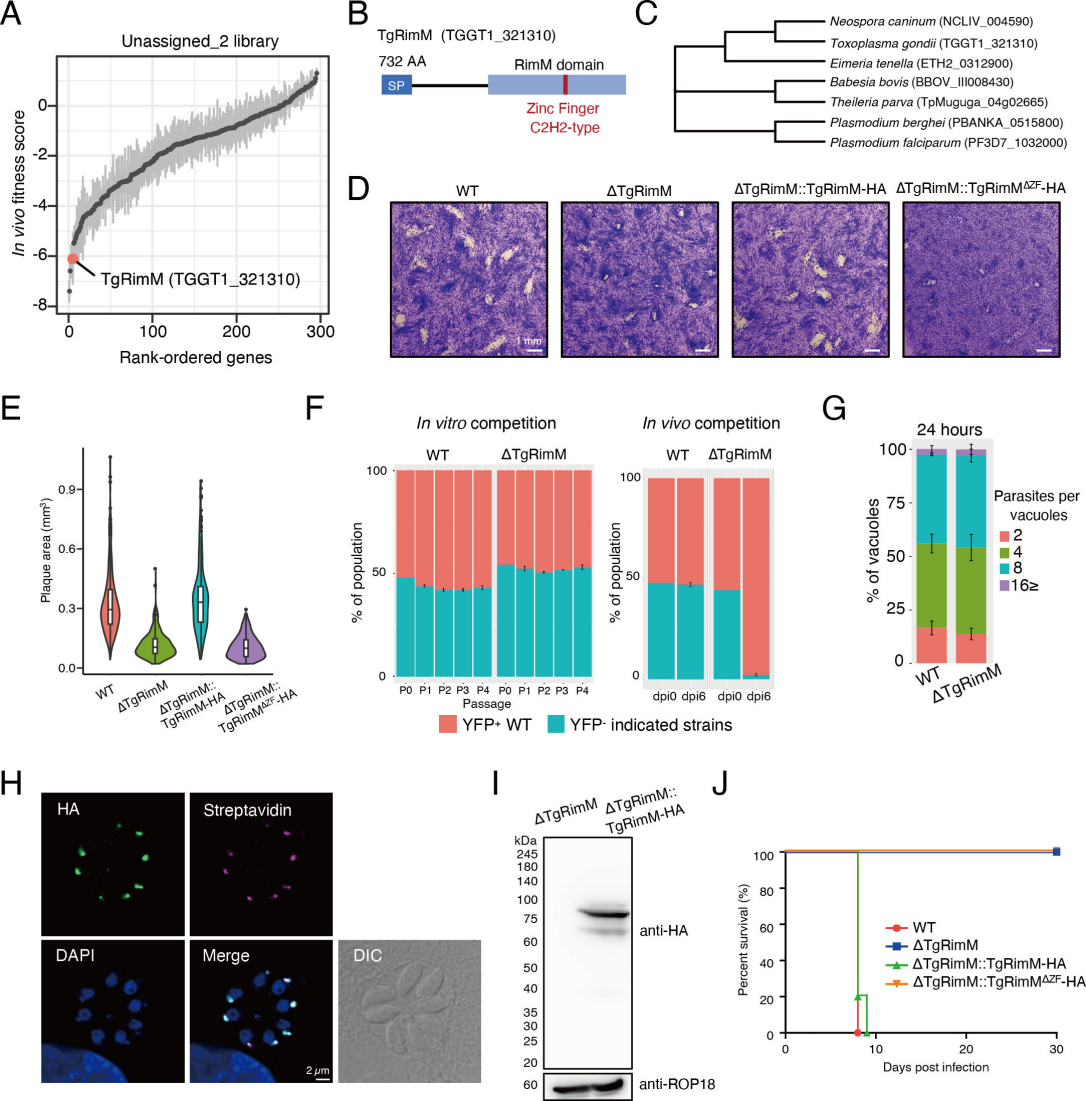
TgRimM is an apicoplast-resident protein that is essential for parasite virulence. (A) Rank-ordered plot of genes from Unassigned_2 library. (B) Schematic of putative domains in TgRimM. SP is predicted by SignalP 6.0. (C) Phylogenetic tree of selected TgRimM homologs across the apicomplexan phylum. (D) Plaque images of indicated strains. (E) Plaque areas of indicated strains. (F) *In vitro* and *in vivo* competition assay of ∆TgRimM and WT. (G) Intracellular replication assay of ∆TgRimM and WT. (H) Immunofluorescence images of ΔTgRimM::TgRimM-HA showing TgRimM-HA (green) is co-stained with streptavidin (magenta), an apicoplast marker. (I) Western blot analysis of TgRimM-HA. (J) Survival curves of C57BL/6 mice intraperitoneally infected with 10^3^ tachyzoites of WT (*n* = 5), ΔTgRimM (*n* = 5), ΔTgRimM::TgRimM-HA (*n* = 5), or ΔTgRimM::TgRimM^ΔZF^-HA (*n* = 5). Data are representative of two (D, E, F, and G) and three (H and I) independent experiments.

Among the top-ranking candidates from Unassigned_2 library, we chose to characterize TgRimM further (Fig. 4A; Table S2). TgRimM possesses a signal peptide, a ribosome maturation factor RimM domain, and a single C2H2-type zinc finger domain (Fig. 4B) (33, 34). We searched for TgRimM homologs and found that they are broadly conserved across the phylum Apicomplexa except for *Cryptosporidium* spp. (Fig. 4C). We performed plaque assays to examine whether deletion of TgRimM affects *in vitro* growth. We found that ∆TgRimM parasites formed smaller plaques compared to WT parasites (Fig. 4D and E), indicating that the deletion of TgRimM impaired *in vitro* growth. To assess the importance of TgRimM in fitness, we performed competition assays in *in vitro* culture (Fig. 4F). ∆TgRimM parasites did not show a significant defect in *in vitro* fitness until the fourth passage. Next, we performed competition assays for *in vivo* infection (Fig. 4F). In contrast to *in vitro* competition, we found that ∆TgRimM parasites were significantly outcompeted by WT parasites in mice. Our results suggest that TgRimM is also important in the *in vivo* environment. To investigate each step of the lytic cycle, we assessed parasite invasion, intracellular replication, egress, attachment, and gliding motility. We found that TgRimM deletion did not affect those events (Fig. 4G; Fig. S4A through D).

To characterize TgRimM further, we complemented the ∆TgRimM strain with C-terminal HA-tagged TgRimM expressed from the tubulin promoter that we will refer to as “∆TgRimM::TgRimM-HA” (Fig. S4E). ∆TgRimM::TgRimM-HA parasites restored normal plaque formation (Fig. 4D). The HA-tagged TgRimM showed an apicoplast localization co-stained with streptavidin (an apicoplast marker) by IFA (Fig. 4H). Western blot against TgRimM-HA showed the main band at approximately 75 kDa, probably a processed form of TgRimM (Fig. 4I). To check the effect of TgRimM on virulence, we infected C57BL/6 mice intraperitoneally with 10^3^ tachyzoites of the WT, ∆TgRimM, and ΔTgRimM::TgRimM-HA parasites and assessed the mouse survival. All the mice intraperitoneally infected with ∆TgRimM parasites survived. In contrast, ΔTgRimM::TgRimM-HA parasites restored virulence (Fig. 4J). Next, we assessed the importance of the C2H2-type zinc finger domain in TgRimM. We generated a complemented strain lacking the C2H2 zinc finger (ΔTgRimM::TgRimM^ΔZF^-HA) (Fig. S4E). Deleting the zinc finger domain did not alter the apicoplast localization of TgRimM^ΔZF^-HA (Fig. S4F). However, ΔTgRimM::TgRimM^ΔZF^-HA could not restore normal plaque formation or virulence (Fig. 4D, E and J). Thus, these results suggest that TgRimM is essential for *in vitro* and *in vivo* growth and that the C2H2 zinc finger is indispensable for TgRimM function in the apicoplast.

## DISCUSSION

Despite much effort from the research community, many apicomplexan proteins still need to be annotated owing to their uniqueness and absence in other model organisms. The CRISPR screens enabled the researchers to investigate hundreds to thousands of genes in *Toxoplasma* at once in *in vitro* culture or *in vivo* infection to identify essential genes for parasite growth, metabolism, drug resistance, differentiation, host–pathogen interaction, and virulence (10–15, 25, 35–44). Most *in vivo* CRISPR screens in *Toxoplasma* have been focused on identifying secreted virulence effectors such as rhoptry bulb proteins (ROPs) and dense granule proteins (GRAs) (11–13). However, recent studies are in progress to search for non-secreted virulence factors by *in vivo* CRISPR screens (14, 15). In this study, we focused on the hyperLOPIT (TAGM-MAP)-unassigned proteins and performed *in vivo* CRISPR screens to identify novel virulence factors in *Toxoplasma*. As a result, we identified several candidate genes that were required for the parasite fitness in mice and further characterized TgGTPase and TgRimM among them.

Although ∆TgGTPase showed a mild *in vitro* growth defect and a severe *in vivo* growth defect, we could not reveal the underlying mechanisms. TgGTPase was originally reported as an interactor of the Nd complex, which plays an important role in rhoptry secretion (19). When we assessed rhoptry secretion of ΔTgGTPase parasites by measuring ROP16-mediated STAT6 phosphorylation and ROP18-dependent Irgb6 coating on PVM, they were comparable to those in WT parasites. Although these data suggest that TgGTPase is not essential for ROP16 or ROP18 secretion, this does not necessarily mean that TgGTPase is not involved in the secretion of all rhoptry proteins. Rather, we cannot rule out the possibility that TgGTPase may control the secretion of rhoptry proteins, other than ROP16 and ROP18, and critically affect virulence. TgGTPase homologs are widely conserved across the phylum Apicomplexa, suggesting that this GTPase is functionally important across the species in the phylum. In the transposon mutagenesis screen in *Plasmodium falciparum* (45), PfGTPase (PF3D7_1348100) possesses lower mutagenesis scores (mutagenesis index score = 0.316 and mutagenesis fitness score = −2.437), suggesting that PfGTPase is also essential for optimal growth of asexual blood stages *in vitro*. Future studies on TgGTPase homologs in *Plasmodium* or *Cryptosporidium* will reveal their conserved function in the phylum.

We identified TgRimM, a novel apicoplast-resident protein essential for normal *in vitro* and *in vivo* growth. A homolog in *P. falciparum* (PfRimM:PF3D7_1032000) is also predicted to be localized in the apicoplast by signal peptide prediction (46). In the transposon mutagenesis screen in *P. falciparum* (45), PfRimM also possesses very low mutagenesis scores (mutagenesis index score = 0.158 and mutagenesis fitness score = −2.8), indicating that PfRimM is essential for growth of asexual blood stages *in vitro*. Assuming that RimM homologs in the phylum Apicomplexa (ApiRimM) are located in the apicoplast, the lack of RimM homologs in *Cryptosporidium* is reasonable because *Cryptosporidium* spp. are known to have lost the apicoplast (47–49). RimM domain-containing protein was reported to regulate 16S rRNA processing in bacteria (50). It is reported that a RimM domain-containing protein in *Arabidopsis thaliana* (AtRimM) is located in the chloroplasts and contributes to rRNA maturation and proteostasis (51). It is considered that the apicoplast evolved from secondary endosymbiosis (52). Considering that the zinc finger domain is widely conserved among ApiRimM (53) and the zinc finger of TgRimM is essential for its function, ApiRimM might facilitate rRNA maturation and proteostasis in the apicoplast via its zinc finger domain. Previous studies on *Toxoplasma* apicoplast have shown that apicoplast function is essential for the parasite *in vitro* survival and growth (54–56). Although ∆TgRimM showed modest *in vitro* and severe *in vivo* growth defects, we could not reveal the underlying mechanisms. We also observed a discrepancy between defects in plaque formation and normal *in vitro* fitness in competition assays. This might arise from their differences in experimental conditions (undisturbed for 7 days vs. passaged every 3 days). Considering that TgRimM is an apicoplast-resident protein, one possibility is the depletion of essential nutrients from the growth medium, such as fatty acids, isoprenoids, heme, or iron-sulfur cluster. Pooled CRISPR screens maintain thousands of mutants in the same culture or infection, and the fitness score is the relative abundance of each mutant. Thus, the pooled CRISPR screen is likely closer to a competition assay rather than a plaque assay. We speculate that many knockout parasites with neutral *in vitro* fitness scores could exhibit small plaques.

Limitations of our current study are that, although 1,198 proteins are classified as unassigned subcellular localizations in hyperLOPIT, our sublibraries cover only half of them. Therefore, the remaining proteins still need to be addressed in the future. Also, although we identified previously unreported genes essential for *in vivo* virulence in this study, their precise functions and localizations mostly remained unaddressed. Future works will aim to characterize these remaining proteins further. *In vivo* CRISPR screens in mice with different genetic backgrounds, such as *Ifngr1^−/^*^−^ mice, may reveal the aspects of these uncharacterized genes.

In conclusion, our *in vivo* CRISPR screen platform provides a resource for the community to identify genes essential for *in vivo* virulence encoding proteins with previously unknown localizations and functions.

## MATERIALS AND METHODS

### *Toxoplasma* strains

RHΔhxgprt (14), RHΔhxgprtΔku80 (57), and its derivatives of *Toxoplasma* were maintained in Vero cells and passaged every 3 days using RPMI (Nacalai Tesque) supplemented with 2% heat-inactivated fetal bovine serum (FBS; JRH Bioscience), 100 U/mL of penicillin and 0.1 mg/mL of streptomycin (Nacalai Tesque) in incubators at 37℃ and 5% CO_2_. ∆ROP16 (58), ∆ROP18 (14), and ∆GRA17 (14) parasites were described previously.

### Host cell culture

Vero cells were maintained in RPMI (Nacalai Tesque) supplemented with 10% heat-inactivated FBS, 100 U/mL of penicillin, and 0.1 mg/mL of streptomycin (Nacalai Tesque) in incubators at 37℃ and 5% CO_2_. Human foreskin fibroblasts (HFFs) and mouse embryonic fibroblasts (MEFs) were maintained in Dulbecco’s modified Eagle’s medium (DMEM) (Nacalai Tesque) supplemented with 10% heat-inactivated FBS, 100 U/mL of penicillin, and 0.1 mg/mL of streptomycin (Nacalai Tesque) in incubators at 37℃ and 5% CO_2_.

### Mice

C57BL**/**6NCrSlc (C57BL/6N) mice were purchased from SLC. All experiments were conducted in 8- to 10-week-old female mice.

### *In vitro* and *in vivo* pooled CRISPR screens

The gRNA sequences of Unassigned_1 and Unassigned_2 sublibrary were selected from the genome-wide gRNA library (10). The selected gRNA sequences were cloned into the modified pU6-Universal vector by cloning a T2A, DHFR, T2A, and RFP in frame with Cas9, where the expression of gRNA and Cas9-T2A-DHFR-T2A-RFP cassettes was independently transcribed. The insertion of the selected gRNA sequences into the vector was performed by VectorBuilder. The gRNA library (200 µg) was linearized with NotI and transfected into approximately 1–2 × 10^8^ RHΔhxgprt parasites divided between four separate cuvettes. Then, transfected parasites were grown in 4 × 150-mm dishes with confluent Vero cell monolayers. Pyrimethamine (Sigma) was added 24 h post transfection. All the parasites were passaged every 3 days until passage 3 without filtration. After 2 days (passage 4), the parasites were syringe lysed, filtered, and counted for genomic DNA preparation or for mouse infection. For genomic DNA preparation, at least 1 × 10^8^ parasites were pelleted and stored at −80°C. For mouse infection, the parasites were resuspended in phosphate-buffered saline (PBS) at a concentration of 2.5 × 10^8^ parasites/mL. Then, 1 × 10^7^ parasites in 40  µL of PBS were injected into the footpad of each anesthetized mouse. Parasite viability was determined by plaque assay. At 7 days post infection, the spleens were collected and crushed by a plunger and passed through a cell strainer to make single-cell suspensions. Then, the suspensions were pelleted and added to 2 × 150-mm dishes per spleen with confluent Vero cell monolayers. After 2–4 days, when the parasites completely lysed out, they were filtered and counted. At least 1 × 10^8^ parasites were pelleted and stored at −80°C. Parasite genomic DNA was extracted using the DNeasy Blood and Tissue kit (Qiagen) according to the manufacturer’s instructions. Integrated gRNA sequences were PCR amplified and barcoded with KOD FX Neo (TOYOBO) using Primer 1 and Primer 2 (Table S3). Genomic DNA (1 µg) was used for the template. The resulting libraries were sequenced on a DNBSEQ-G400RS (MGI) using Primer 3 and Primer 4 (Table S3).

### Bioinformatic analysis of the CRISPR screen

Following demultiplexing, gRNA sequencing reads were aligned to the gRNA library. The abundance of each gRNA was calculated and normalized to the total number of aligned reads (59). For *in vitro* analysis, the log_2_ fold change between the P4 sample and the library was calculated for each gRNA. The fitness score for each gene was calculated as the mean log_2_ fold change for the top five scoring guides. For *in vivo* analysis, the log_2_ fold change between each *in vivo* sample and the P4 sample was calculated for each gRNA as described above. The median fitness score across mouse replicates was used as the *in vivo* fitness score (14). For a given gene, gRNAs were compared using Wilcoxon rank sum test between P4 vs WT mice. The *P* values for each test were adjusted using the Benjamini–Hochberg method. The distance of each gene from the regression line was calculated as below.


$$distance\;\left(ax+by+c=0,\left(x_{0},y_{0}\right)\right)=\frac{ax_{0}+b\;y_{0}+c}{\sqrt{a^{2}+b^{2}}}$$

All analyses were conducted by R (v4.1.1) with package stats (v3.6.2) and visualized by ggplot2 (v3.4.0).

### Plasmid construction for knockout *Toxoplasma*

For construction of the CRISPR/Cas9 plasmids for targeting a gene of interest (GOI), two oligonucleotide primers (GOI_gRNA1_F and GOI_gRNA1_R, GOI_gRNA2_F and GOI_gRNA2_R) containing gRNA sequence were annealed and cloned into BsaI site of the pU6-Universal vector (Addgene #52694). To generate a construct for deleting the entire coding region of GOI, flanking regions of 60 bp of 5′ and 3′ outside the gRNAs were used to surround the genes. Forward and reverse primers with homology to the floxed HXGPRT cassette and to the 5′ and 3′ coding sequence of the GOI was used (GOI_homology_F and GOI_ homology _R). The primer sequences used for the genetic disruption are shown in Table S3.

### Generation of gene knockout *Toxoplasma*

RHΔhxgprtΔku80 was filtered and resuspended in Cytomix (10 mM KPO_4_, 120 mM KCl, 0.15 mM CaCl_2_, 5 mM MgCl_2_, 25 mM HEPES, 2 mM EDTA). Parasites were mixed with 50 µg each of gRNA1 and gRNA2 CRISPR plasmids with the PCR-amplified targeting fragment for each GOI, and supplemented with 2 mM ATP, 5 mM GSH. Parasites were electroporated by GENE PULSER II (Bio-Rad Laboratories). Transfected parasites were selected by 25 µg/mL of mycophenolic acid (MPA) (Sigma) and 50 µg/mL of xanthine (Wako) to obtain stably resistant clones. Then, parasites were subjected to limiting dilution in 96-well plates to isolate single clones. To confirm the disruption of the gene, we analyzed the mRNA expression by quantitative RT-PCR.

### Complementation of TgGTPase-HA and TgRimM

To complement the knockout parasites, TgGTPase, TgRimM, and TgRimM^ΔZF^ cDNA were generated by PCR amplification from cDNA of the RH strain. The PCR products were inserted into the pUPRT plasmid vector, which possesses the tubulin promoter and C-terminal HA tag. Knockout parasites were transfected with the pUPRT complementation vector and gRNA-targeting uracil phosphoribosyltransferase (UPRT) locus, then selected by fluorodeoxyuridine (FUDR) (Wako) (60). Parasites were subjected to limiting dilution in 96-well plates. Isolated clones were examined for protein expression by western blotting and IFA.

### Complementation of HA-TgGTPase

To complement the ΔTgGTPase parasites, HA-TgGTPase cDNA was generated by PCR amplification from cDNA of the RH strain. The PCR products were inserted into the plasmid vector that possesses HXGPRT expression cassette and the SAG1 promoter. The HXGPRT cassette, which is inserted in the endogenous TgGTPase locus of ∆TgGTPase parasites, was deleted with Cre transfection and selected in the presence of 6-thioxanthine (6-TX). ∆TgGTPase∆hxgprt parasites were transfected with the HA-TgGTPase complementation vector and selected in the presence of MPA/Xanthine and subjected to limiting dilution. Isolated clones were examined for HA-TgGTPase protein expression by western blotting.

### TgGTPase and TgRimM phylogenetic analysis

ToxoDB (33), VEuPathDB (53), and BLAST were searched for TgGTPase or TgRimM homologs across the phylum Apicomplexa. Clustal Omega was used to align obtained homologs (61). Phylogenetic trees were visualized by ggtree (v3.2.1) (62).

### Immunofluorescence assay

HFFs were grown on coverslips, infected with parasites for 24–30 h, and fixed in PBS containing 3.7% paraformaldehyde (PFA) for 10 min at room temperature. Cells were permeabilized with PBS containing 0.1% Triton-X or 0.002% Digitonin for 10 min and then blocked with PBS containing 3% bovine serum albumin (BSA) for 1 h at room temperature. Then, the coverslips were incubated with the primary antibodies for 1 h, followed by incubation with appropriate secondary antibodies, streptavidin, and 4′,6-diamidino-2-phenylindole (DAPI) for 30 min. The coverslips were mounted using PermaFluor (Thermo Scientific). Images were acquired by confocal laser microscopy (Olympus FV3000 IX83). Primary antibodies used were mouse anti-HA.11 (BioLegend, 901514), rabbit anti-GRA17 (14), mouse anti-SAG1 (63), rabbit anti-GAP45 (63), rabbit anti-phospho-STAT6 (Cell Signaling, #9361), goat anti-Irgb6 (Santa Cruz, sc-11079), and Alexa Fluor 594 streptavidin (Invitrogen, S11227).

### Assessment of *in vivo* virulence in mice

Mice were infected with 10^3^ tachyzoites in 200 µL (intra-peritoneum) or 40 µL (intra-footpad) of PBS. Parasite viability was determined by plaque assay. The mouse health condition and survival were monitored daily until 30 days (intra-peritoneum) or 40 days (intra-footpad) post infection, respectively.

### Plaque assay

A total of 400 parasites were infected into a well of a six-well plate of HFFs and grown for 7 days in DMEM (Nacalai Tesque) supplemented with 5% heat-inactivated FBS. Parasites were fixed with 3.7% PFA, stained with crystal violet, washed, and dried overnight. Plaque areas were measured by ImageJ.

### *In vitro* and *in vivo* competition assay

Competition assays were conducted with YFP-positive WT parasites and YFP-negative indicated strains. YFP-positive and -negative parasites were mixed in a 1:1 ratio, and the exact input ratio was checked by flow cytometry. For *in vitro* competition assay, 3 × 10^5^ mixed parasites were inoculated into 60 mm dishes of Vero cells and passaged every 3 days until fourth passage. For each passage, the parasite ratio was measured by flow cytometry. For *in vivo* competition assay, 5 × 10^3^ mixed parasites in 200 µL of PBS were injected intraperitoneally to mice. At 6 days post-infection, parasites were retrieved from peritoneal lavage, filtered, and directly measured by flow cytometry. Data were collected by Attune NxT (Thermo Fisher) and analyzed with FlowJo ver10.

### Invasion assay

Parasites (5 × 10^5^) were inoculated on coverslips with HFF monolayer in a 24-well plate. The plate was centrifuged for 5 min at 250 × *g*, incubated for 30 min at 37°C, and fixed with 3.7% PFA. Extracellular parasites were stained with anti-SAG1 antibody without permeabilization. After washing three times, the cells were permeabilized with 0.1% Triton-X; then, all parasites were stained with anti-GAP45 antibody. At least 100 parasites were counted and determined as extracellular or intracellular parasites.

### Replication assay

A total of 4 × 10^4^ parasites were inoculated on coverslips with HFF monolayer in a 24-well plate. Twenty-four hours post-infection, the monolayers were fixed with 3.7% PFA and processed for IFA with anti-GAP45 antibody to stain parasites. The number of parasites per vacuole were counted for at least 100 vacuoles.

### Induced egress assay

Freshly egressed parasites were inoculated on coverslips with HFF monolayer and grown for 24–30 h at 37°C. After washing with serum-free DMEM twice, the infected monolayers were incubated with 2 µM ionomycin (Nacalai Tesque) in serum-free DMEM for 5 min at 37°C. Cells were fixed with 3.7% PFA and processed for IFA with anti-SAG1 and anti-GRA17 antibody to stain parasites and PVs, respectively. At least 100 vacuoles were counted per strain and scored as occupied or egressed.

### Gliding motility assay

Freshly egressed parasites were resuspended in serum-free DMEM and added to poly-L-lysine-coated coverslips in a 24-well plate. The plate was centrifuged for 3 min at 250 × *g*. The media were replaced with serum-free media containing 2% EtOH. Following incubation for 30 min at 37°C, the coverslips were fixed with 3.7% PFA and processed for IFA with anti-SAG1 antibody to stain SAG1 trails.

### Attachment assay

Freshly egressed YFP-positive WT parasites and YFP-negative indicated strains were mixed in a 1:1 ratio in intracellular (IC) buffer (5 mM NaCl, 142 mM KCl, 1 mM MgCl_2_, 2 mM EGTA, 5.6 mM glucose, 25 mM HEPES, pH adjusted to 7.2 with KOH) containing 1 µM cytochalasin D (CytD). The exact input ratio was checked by flow cytometry. Following 10 min of incubation at room temperature, parasites were added to HFF-coated coverslips in a 24-well plate. The plate was centrifuged for 2 min at 250 × *g*. The medium was replaced with warm DMEM with 5% FBS and 1 µM CytD. The plate was incubated for 20 min at 37°C and fixed with 3.7% PFA. IFA was performed using anti-SAG1 antibody to stain all parasites. The ratio between SAG1-positive (all parasites) and YFP-positive parasites was counted and normalized by the input ratio.

### ROP16-mediated STAT6 phosphorylation assay

A total of 5 × 10^5^ parasites were inoculated on coverslips with HFF monolayer in a 24-well plate. The plate was centrifuged for 5 min at 250 × *g*, incubated for 30 min at 37°C, and fixed with cold methanol for 10 min. The cells were stained with anti-pSTAT6 and anti-SAG1 antibody overnight at 4°C. At least 100 cells were counted and determined as pSTAT6-positive nuclei or not.

### ROP18-dependent Irgb6 recruitment assay

MEFs were seeded on glass coverslips and stimulated with 10  ng/mL of IFN-γ for 24 h. The cells were infected with parasites at a multiplicity of infection (MOI) of 4 and incubated for 2 h at 37°C. The cells were stained with anti-Irgb6 and anti-GRA17 antibody. At least 100 vacuoles were counted and determined as Irgb6-positive PVs or not.

### Quantitative RT-PCR

Total RNA was extracted by RNeasy kit (QIAGEN), and cDNA was synthesized by Verso reverse transcription (Thermo Fisher Scientific) according to the manufacturer’s instructions. Quantitative RT-PCR was performed with a CFX Connect real-time PCR system (Bio-Rad Laboratories) and a Go-Taq real-time PCR system (Promega). The data were analyzed by the ∆∆CT method and normalized to ACT1 in each sample. The primer sequences are listed in Table S3.

### Western blotting

Cells were lysed in lysis buffer (1% NP-40, 150  mM NaCl, 20  mM Tris-HCl, pH 7.5) containing a protease inhibitor cocktail (Nacalai Tesque). The cell lysates were separated by SDS-PAGE and transferred to polyvinylidene difluoride membranes (Immobilon-P; Millipore). Membranes were blocked in 5% skim milk in 0.05% Tween-20 in PBS for 1 h at room temperature, followed by incubation with primary antibodies for 1 h at room temperature. Blots were then incubated with appropriate secondary antibodies for 1 h at room temperature. Primary antibodies used were mouse anti-HA (MBL, M180-7) and rabbit anti-ROP18 (this study).

### Generation of custom anti-ROP18 antibody

Custom anti-ROP18 antibody (rabbit polyclonal) was generated against a synthetic C-terminal peptide of ROP18 (AQNFEQQEHLHTE). The epitope identification, peptide synthesis, rabbit immunization, and serum collection were conducted by Cosmo Bio. The specificity of anti-ROP18 antibody was validated for western blotting.

### Quantification and statistical analysis

Information about the number of biological replicates and the type of statistical tests used can be found in the figure legends. All statistical analyses except for the survivals were performed using R (4.1.1, https://www.r-project.org/). For correlation analysis, Pearson’s correlation was used. Data with *P* values < 0.05 were considered statistically significant. The statistical analysis of survival rates was performed by the log-rank test using the GraphPad Prism9 software.

## Data Availability

The CRISPR screen data of two sublibraries have been deposited to the NCBI GEO. The GEO accession numbers are GSE253884 and GSE253885. Any additional information required to reanalyze the data reported in this paper is available from the corresponding author upon request.

## SUPPLEMENTAL MATERIAL

Supplemental figures mbio.01728-24-s0001.pdf Figures S1 to S4.

Table S1 mbio.01728-24-s0002.xlsx Summary of *in vivo* CRISPR screen using Unassigned_1 library, gRNA sequences, raw count data, and *in vivo* fitness scores for each mouse.

Table S2 mbio.01728-24-s0003.xlsx Summary of *in vivo* CRISPR screen using Unassigned_2 library, gRNA sequences, raw count data, and *in vivo* fitness scores for each mouse.

Table S3 mbio.01728-24-s0004.xlsx Primers used in this study.

ASM does not own the copyrights to Supplemental Material that may be linked to, or accessed through, an article. The authors have granted ASM a non-exclusive, world-wide license to publish the Supplemental Material files. Please contact the corresponding author directly for reuse.
