# Supplementary material for: CRISPR screens identify genes essential for in vivo virulence among proteins of hyperLOPIT-unassigned subcellular localization in Toxoplasma
Source: mBio. 2024 Jul 31;15(9):e01728-24. doi: 10.1128/mbio.01728-24 (PMC11389413; doi:10.1128/mbio.01728-24)
Supplement: Supplemental figures — Figures S1 to S4. [file mbio.01728-24-s0001.pdf]

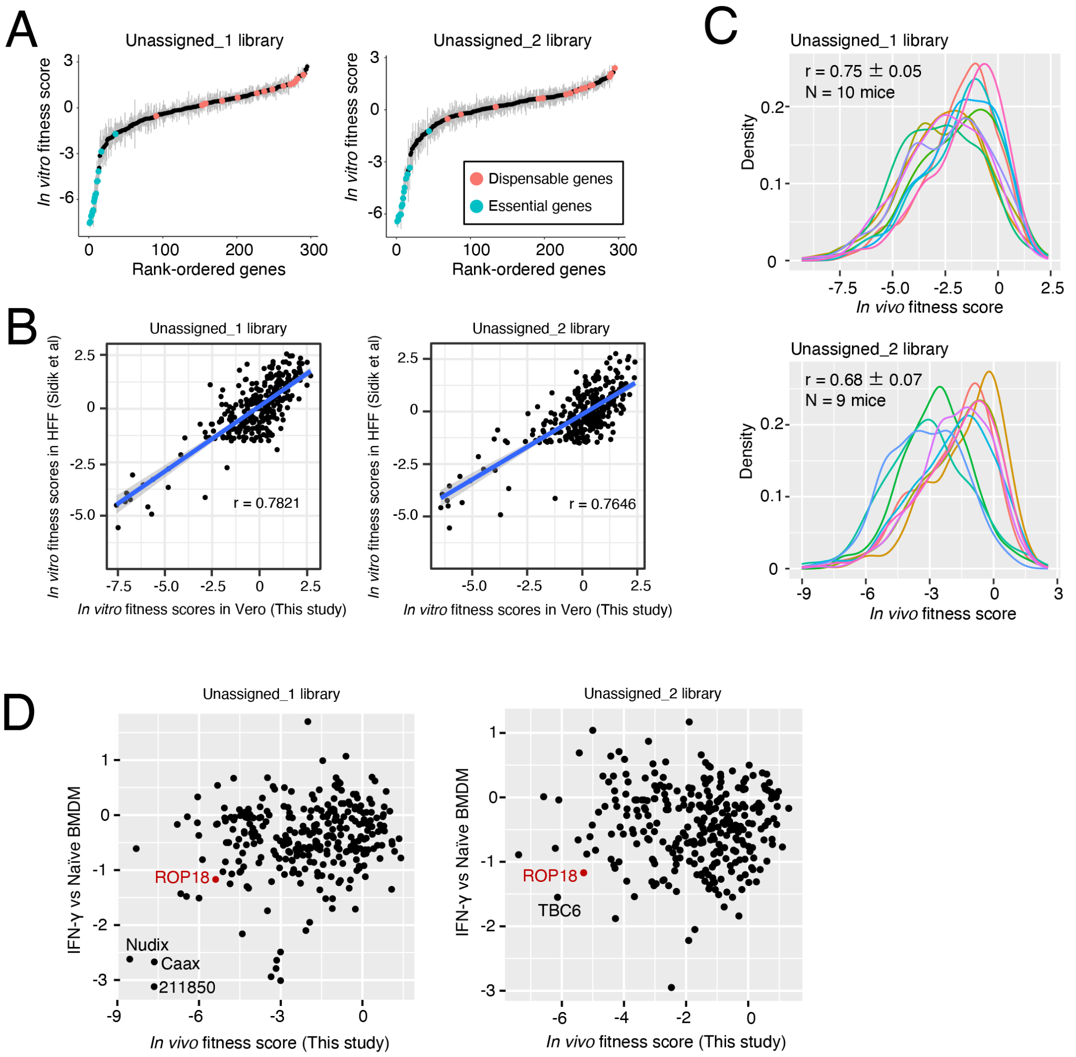

Supplementary Figure 1. Assessing the reproducibility of CRISPR screens. Related to Figure 1.

(A) Rank-ordered plots for *in vitro* fitness scores of 4th passage.

(B) Correlation between our *in vitro* fitness scores and the *in vitro* fitness scores in HFF.

(C) Overlay of *in vivo* fitness scores for each mouse. Pearson's correlation coefficients are shown as mean ± SD.

(D) Scatter plots comparing fitness scores from *in vivo* screens (this study) and IFN-γ-stimulated macrophages. ROP18, a known IFN-γ-dependent fitness gene, is highlighted in red.

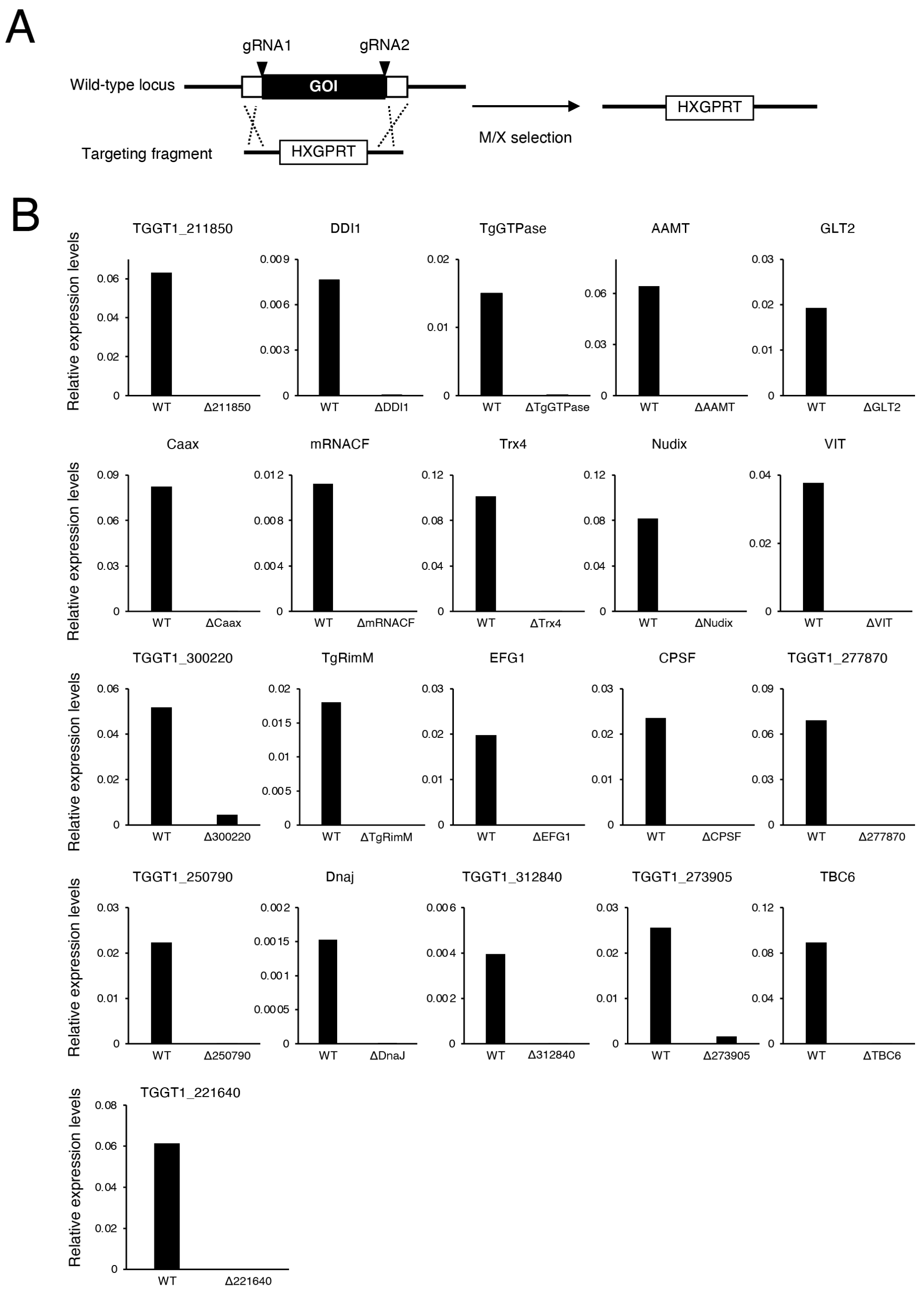

Supplementary Figure 2. Generating gene knockout parasites. Related to Figure 2.

(A) Schematic of gene knockout strategy.

(B) Quantitative RT-PCR validations for indicated parasites.

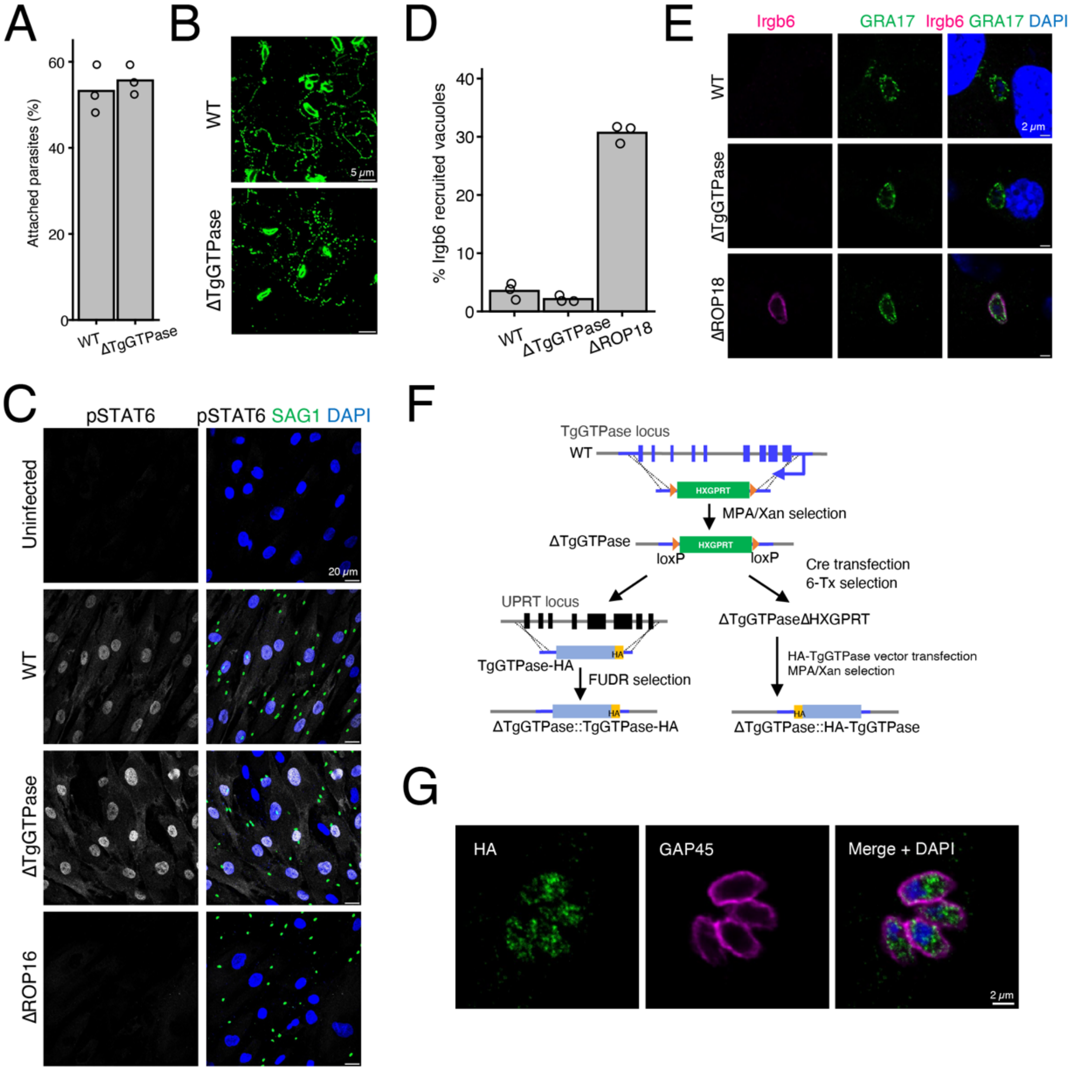

Supplementary Figure 3. Deletion of TgGTPase does not affect parasite attachment, gliding motility, or rhoptry secretion.

Related to Figure 3.

(A) Attachment assay of ΔTgGTPase and WT.

(B) Gliding motility assay of ΔTgGTPase and WT. Representative images are shown.

(C) Immunofluorescence images of HFFs uninfected or infected with indicated strains. Cells were stained with anti-pSTAT6 (white), SAG1 (green), and DAPI.

(D) Irgb6 recruitment assay of indicated strains.

(E) Immunofluorescence images of MEFs infected with indicated strains. Cells were stained with anti-Irgb6 (magenta), GRA17 (green), and DAPI.

(F) Schematic of TgGTPase knockout and complementation.

(G) Immunofluorescence images of ΔTgGTPase::HA-TgGTPase showing cytosolic localization of HA-TgGTPase (green).

Data are representative of two (A, B, D, E, and G) and three (C) independent experiments.

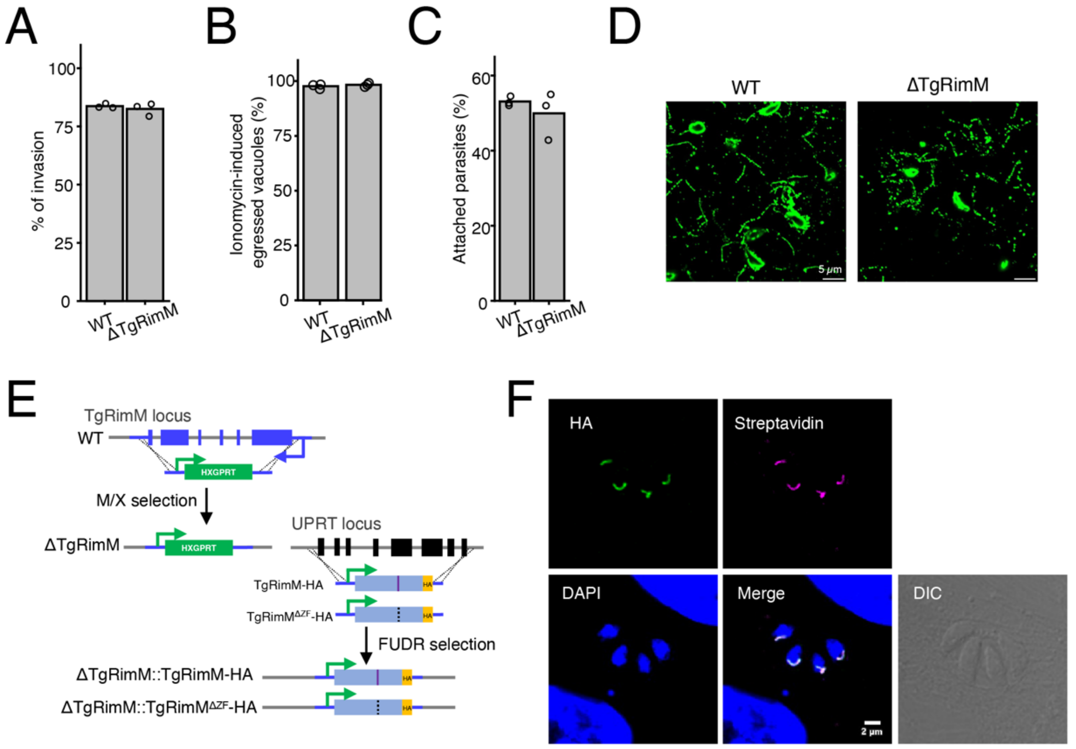

Supplementary Figure 4. Deletion of TgRimM does not affect parasite invasion, egress, attachment, or gliding motility.

Related to Figure 4.

(A) Invasion assay of ΔTgRimM and WT.

(B) Induced egress assay of ΔTgRimM and WT.

(C) Attachment assay of ΔTgRimM and WT.

(D) Gliding motility assay of ΔTgRimM and WT. Representative images are shown.

(E) Schematic of TgRimM knockout and complementation.

(F) Immunofluorescence images of ΔTgRimM::TgRimM$^{\Delta ZF}$-HA showing TgRimM$^{\Delta ZF}$-HA (green) is co-stained with streptavidin (magenta), an apicoplast marker.

Data are representative of two (A, B, C, and D) and three (F) independent experiments.

Table S1.

Summary of *in vivo* CRISPR screen using Unassigned_1 library, gRNA sequences, raw count data, and *in vivo* fitness scores for each mouse.

Table S2.

Summary of *in vivo* CRISPR screen using Unassigned_2 library, gRNA sequences, raw count data, and *in vivo* fitness scores for each mouse.

Table S3.

Lists of the primers used in this study.
